# A Case of Autoimmune Hemolytic Anemia after the First Dose of COVID-19 mRNA-1273 Vaccine with Undetected Pernicious Anemia

**DOI:** 10.1155/2022/2036460

**Published:** 2022-01-29

**Authors:** Fnu Jaydev, Vinod Kumar, Jaikumar Khatri, Shobha Shahani, Sead Beganovic

**Affiliations:** ^1^Division of Internal Medicine and Geriatrics, Indiana University School of Medicine, Indianapolis, IN, USA; ^2^Indiana University Health Central Indiana Cancer Center, Indianapolis, IN, USA

## Abstract

By this time, multiple vaccines have been approved to limit the spread of SARS-CoV-2 worldwide. These include new-generation vaccines that contain mRNA of the target organism. Some common side effects were identified and reported during phase 3 clinical trials of vaccination, but more rare adverse events were reported in the literature. One such concern is autoimmune conditions that SARS-CoV-2 viral antigens could have possibly incited. We are presenting here a case of a young female with no known autoimmune diseases, diagnosed with autoimmune hemolytic anemia about a week after receiving her first dose of the COVID-19 mRNA vaccine. We discuss the possible culprit for precipitation of autoimmune hemolytic anemia after the SARS-CoV-2 mRNA vaccine, which encodes virus spike protein. This case highlights the importance of being vigilant for identifying rare adverse events that could appear during mass vaccination.

## 1. Introduction

Since the beginning, the SARS-CoV-2 pandemic has claimed many lives worldwide and caused significant morbidity in many patients [1]. Besides the usual systemic symptoms and signs of COVID-19 infection, some rare medical conditions have been reported in the literature. One such rare phenomenon which has been reported in the literature is autoimmune conditions such as Guillain–Barré syndrome [2], immune thrombocytopenic purpura [3], antiphospholipid antibody syndrome [4], and autoimmune hemolytic anemia, both warm antibody type and cold agglutinin type [4–7]. The first effective approved vaccines against the SARS-CoV-2 virus were the mRNA vaccines from two major pharmaceutical companies, to be followed by mass vaccination programs in many parts of the world [8]. Some of the common side effects of these vaccines include local injection site inflammation, fever, chills, fatigue, myalgia, arthralgia, and headache [9], which are primarily self-resolving within a couple of days. Some relatively rare side effects were reported as medically attended adverse events (MAAEs); for example, Bell's palsy and severe adverse events (SAEs) were reported during clinical trials of the COVID-19 mRNA-1273 vaccine. Despite the numerical difference in frequency of these events, no causal relationship could be established, given the small number of cases. We present a case of warm antibody autoimmune hemolytic anemia in a female with the onset of symptoms about a week after receiving her first COVID-19 mRNA-1273 vaccine shot, who was later found to have undetected pernicious anemia as well.

## 2. Case

The case was a 42-year-old female with a past medical history of hypertension, congenitally deaf and mute (uses sign language for communication), iron deficiency anemia due to menorrhagia, and history of provoked venous sinus thrombosis in 2015 treated with anticoagulation, but this had to be stopped due to worsening menorrhagia, without recurrence of venous sinus thrombosis. We obtained history with the help of a sign language interpreter. She was admitted to our hospital with a two-week history of worsening dizziness and lightheadedness, progressive shortness of breath with activity, palpitations, mild spells of blurred vision, generalized weakness, lack of energy, and easy fatigue with her activities at home and her workplace. The patient reported the onset of these symptoms was a week after getting her first dose of the COVID-19 mRNA-1273 vaccine on April 7, 2021. Since then, her symptoms had been progressive and got severe enough to limit her physical activity in the last few days. She reported no cough, fever, nausea, vomiting, abdominal pain, melena, blood in the stools, hematemesis, or urinary symptoms. The patient has been on oral iron supplementation, to which she has been very compliant. As recently checked by her primary physician, her iron level had been stable (ferritin: 32.2 ng/mL in 2016 and 355.4 ng/mL on this admission with serum iron 95 *μ*g/dL and iron saturation of 41%). Her menorrhagia has been stable; her periods last for seven days, with the first four days being a little heavier. Her hemoglobin on admission was found to be 4.5 g/dL, MCV was 90 fL, and absolute reticulocyte count was 19.9 × 10^9^/L (see Table 1 for detailed lab results). Creatinine was within the normal range. She had mildly elevated indirect bilirubin. A peripheral blood smear was reported to show a few schistocytes, polychromasia, and anisocytosis. Haptoglobin was <6 mg/dL. LDH was 4332 IU/L, and the indirect Coombs' test was reported positive. We performed a direct Coombs' test, which was reported positive for IgG and C3 with identification of warm autoantibodies. COVID-19 PCR test was found to be negative.

**Figure 1:**
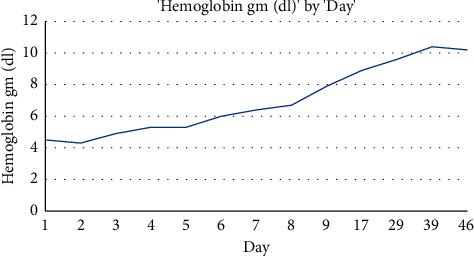
Hemoglobin trend during hospitalization and after discharge.

On further investigations, she was noted to have normal serum iron and iron saturation but mildly elevated ferritin, normal folate, and severely low vitamin B12 level <50 pg/mL. Further testing showed positive gastric parietal cell IgG antibodies and intrinsic factor antibodies. Given the hemodynamic stability of the patient and the patient's preference, she did not receive a blood transfusion. The patient was monitored inpatient and started on intravenous steroids, methylprednisolone (2 mg/Kg daily), and intramuscular cyanocobalamin injections daily. Hemoglobin improved from 4.5 g/dL on admission to 6.0 g/dL on day 7 of admission, and on day 10 (Figure 1: trend of hemoglobin during and after hospitalization), which was the day of her discharge, hemoglobin had improved to 7.9 g/dL with schistocytes still present. Her symptoms and hemoglobin level slowly improved, and she was discharged on a long taper of oral prednisone and continued parenteral cyanocobalamin replacement. On the first follow-up about a week after her discharge, labs showed hemoglobin improvement to 8.9 g/dL with no schistocytes on the peripheral smear. LDH improved to 540 IU/L and absolute reticulocyte to 88.2 × 10^9^/L. On the second follow-up, two weeks later, her hemoglobin improved to 9.6 g/dL (see Table 1).

## 3. Discussion

Autoimmune hemolytic anemia is associated with IgG or IgM antibodies against the intrinsic RBC surface antigens. It causes hemolysis via complement and reticuloendothelial cells, resulting in the extravascular hemolytic process. Our patient was noted to have features of hemolysis (Table 1); both indirect and direct Coombs' tests were positive for warm autoantibodies IgG and C3d. Peripheral blood smear in extravascular hemolysis usually shows spherocytosis, but in this patient, peripheral smear showed moderate to many microcytes and few to moderate macrocytes along with few schistocytes. In autoimmune hemolytic anemia, intravascular hemolysis can be seen depending on the type and concentration of IgG antibodies [10]. We suspect that, in our patient, there could have been a component of intravascular hemolysis along with a predominant extravascular process as evidenced by the presence of few schistocytes and a very high LDH level. The high LDH level, in this case, could also be contributed by the presence of intramedullary hemolytic process secondary to severe vitamin B12 deficiency. Warm antibody AIHA is more commonly associated with lymphoproliferative disorders (e.g., chronic lymphocytic leukemia) and autoimmune disorders (e.g., systemic lupus erythematosus) [11]. Interestingly, our patient was found to have a severe vitamin B12 deficiency; we tested and found her positive for intrinsic factor antibodies and gastric parietal cell antibodies (Table 1), raising the possibility of previously undetected pernicious anemia.

Hemolysis in patients with vitamin B12 deficiency is rare [12] and reported to be 1.5% in a study of hematologic manifestations with vitamin B12 deficiency in 201 patients [13], but the combination of pernicious anemia and autoimmune hemolytic anemia is also an infrequent occurrence. Yeruva et al. reported a case with this rare combination and, from their literature search, found 14 other cases between 1951 and 2010 [14]. In an extensive 18-year study of AIHA in 865 patients, an association of warm antibody AIHA was noted in only 3 cases [15]. Our patient was treated with parenteral cyanocobalamin injection and steroids, resulting in rapid response in hemoglobin, improving from 4.5 g/dl to 9.6 g/dl in less than a month without any red cell transfusion. AIHA in patients with pernicious anemia does not respond to vitamin B12 injections alone but will require steroids [14, 16].

With active hemolysis, one would expect a rapid rise in the reticulocyte count, but our patient's initial reticulocyte count was normal despite signs of severe hemolysis, and we posit that this could be due to intramedullary hemolysis related to vitamin B12 deficiency. Besides intravascular hemolysis, intramedullary hemolysis or ineffective erythropoiesis results in the destruction of red blood cells within the bone marrow and has been recognized as another phenomenon responsible for decreasing red cell counts and anemia. While the exact mechanism is still not well understood, some role of elevated homocysteine level has been postulated as contributing to intramedullary hemolysis based on in vitro experiments and reported in case reports [17–19].

After mass vaccination has rolled out, some rare adverse events have been reported, such as DVT after the BTN162b2 mRNA vaccine [20]. To our knowledge, our case is one of the first few cases of autoimmune hemolytic anemia after the COVID-19 mRNA vaccine, which could have a possible and biologically plausible association with the vaccine. Given the rarity of such disorders, it is hard to make a conclusive argument in favor of the cause-and-effect relationship between the mRNA COVID-19 vaccine and autoimmune hemolytic anemia, but given the previous case reports of AIHA in patients with SARS-CoV-2 infection and recently reported molecular mimicry as a possible culprit in the AIHA-affecting patients with COVID-19 disease, Angileri et al., in their correspondence to the British Journal of Hematology [21], postulated such molecular mimicry between ankyrin-1 (ANK-1), a red cell membrane protein, and SARS-CoV-2 viral spike protein. Using the immune epitope database (IEDB, https://www.iedb.org/), they found a 100% identity between the ANK-1 putative immunogenic-antigenic epitope (amino acids LLLQY) and the spike protein's predicted immunogenic epitope 750-SNLLLQYGSFCTQL-763 for B cells. ANK-1 provides a primary linkage between the membrane skeleton and plasma membrane and, if genetically defective, can result in hemolytic anemia such as hereditary spherocytosis [21, 22]. Since this molecular similarity has been hypothesized as the pathogenic basis for AIHA in patients with SARS-CoV-2, we postulate that the same cross-reactivity can be contributing to AIHA after receiving the COVID-19 mRNA-1273 dose in our case. COVID-19 mRNA-1273 vaccine contains a synthetic messenger ribonucleic acid (mRNA) encoding the prefusion stabilized spike glycoprotein (S) of SARS-CoV-2 virus (2) and induces production of spike protein by human cells, which then results in the production of antibodies against spike protein. These antibodies then cross-react with ANK-1 RBC protein, resulting in autoimmune hemolytic anemia.

Per our literature review, we have found two other reported cases of autoimmune hemolytic anemia associated with the mRNA COVID-19 vaccine [23, 24]. One of these patients [24] had a known history of cold agglutinin disease, which had been stable and got exacerbated by the administration of the COVID-19 vaccine. As per the VRBPAC Briefing Document of the FDA on the COVID-19 mRNA-1273 vaccine, the FDA recommended two months' median follow-up after completion of vaccination which may allow for identification of potential immune-mediated adverse events that began within six weeks of vaccination and could be plausibly linked to vaccination [25].

## 4. Conclusion

The process of warm antibody autoimmune hemolysis in our patient could have an association with underlying undetected pernicious anemia and vitamin B12 deficiency, which in itself is a rare occurrence. Given the timing of presentation of this case and previously reported cases of autoimmune pathologies in association with COVID-19 and with mRNA vaccines, we cannot exclude the possibility of the patient's mRNA vaccination as an inciting or precipitating factor for warm antibody autoimmune hemolysis in this patient. As more and more people worldwide are getting the COVID-19 mRNA vaccine, rare side effects such as autoimmune hemolytic anemia are being reported. Even though rare, these can cause significant morbidity and mortality. This requires high vigilance on the part of the care providers to detect these rare side effects at an earlier stage.

## Figures and Tables

**Table 1:** Laboratory test result during and after hospitalization.

Laboratory results	Admission	Discharge	Follow-up 1 (1 week after discharge)	Follow-up 2 (3 weeks after discharge)	Reference range
WBC (×10^9^/L)	3.5	9.0	11.4	12.2	3.6–10.6
Neutrophils (%)	27	93	86	90	
Lymphocytes (%)	68	7	10	8	
Monocytes (%)	3	0	4	1	
Eosinophils (%)	0	0	0	1	
Basophils (%)	0	0	0	0	
Myelocytes (%)	2				
Absolute neutrophils (×10^9^/L)	0.9	8.4	9.6	11.2	1.7–7.0
Absolute lymphocytes (×10^9^/L)	2.2	0.6	1.1	1.0	1–3.2
Absolute monocytes (×10^9^/L)	0.1	0	0.4	0.1	0.1–1.3
Absolute eosinophils (×10^9^/L)	0	0	0	0.1	0.0–0.3
Absolute basophils (×10^9^/L)	0	0	0	0	0.0–0.2
Absolute myelocytes (×10^9^/L)	0.1				
Hemoglobin (g/dL)	4.5	7.9	8.9	9.6	12–15
Hematocrit (%)	12.7	24.1	27.9	31.2	35–49
MCV (fL)	90	95	93	92	81–99
RDW (%)	>40	39.6	29.2	21.6	11.5–14.5
Platelets (×10^9^/L)	148	401	628	243	150–450
Schistocytes	Few (1+)	Many (3+)	None seen	None seen	None
Polychromasia	Slight	Marked (3+)	Moderate (2+)	Slight	
Microcytes	Many (3+)	Moderate (2+)	Few (1+)		
Macrocytes	Few (1+)	Moderate (2+)	Moderate (2+)	Few (1+)	
ALT (units/L)	36				7–52
AST (units/L)	71				13–39
Total bilirubin (mg/dL)	2.1			0.5	0.0–1.0
Indirect bilirubin (mg/dL)	1.7			0.4	0.0–0.8
LDH (units/L)	4332	2628	540	257	140–271
Haptoglobin (mg/dL)	<6	<6			30–200
Serum iron (*μ*g/dL)	95				50–212
Iron saturation (%)	41				15–55
TIBC (*μ*g/dL)	231				250–400
Ferritin (ng/mL)	355.4				10–106
Serum B12 level (pg/mL)	<50				200–1,000
Serum folate (ng/mL)	>23.0				>5.9
Reticulocyte count (%)	1.4		2.8	2.0	0.5–2.5
Absolute reticulocyte number (× 10^9^/L)	19.9		88.2	69.1	21–115
Gastric parietal cell IgG (units)	18.8				0.0–24.9
Intrinsic factor antibodies	Positive				Negative
Indirect Coombs' test	Positive				Negative
Direct Coombs' test	Positive IgG and C3				Negative
Antibody ID	Warm autoantibodies				Negative
Antibody eluted	Eluate positive with all cells				
Coronavirus SARS-CoV-2 PCR	Not detected				

## Data Availability

The data used to support the findings of this case report are included within the article.

## References

[1] Pollard C. A., Morran M. P., Nestor-Kalinoski A. L. (2020). The COVID-19 pandemic: a global health crisis. *Physiological Genomics*.

[2] Toscano G., Palmerini F., Ravaglia S. (2020). Guillain-barré syndrome associated with SARS-CoV-2. *New England Journal of Medicine*.

[3] Zulfiqar A.-A., Lorenzo-Villalba N., Hassler P., Andrès E. (2020). Immune thrombocytopenic purpura in a patient with covid-19. *New England Journal of Medicine*.

[4] Zhang Y., Xiao M., Zhang S. (2020). Coagulopathy and antiphospholipid antibodies in patients with covid-19. *New England Journal of Medicine*.

[5] Hindilerden F., Yonal-Hindilerden I., Akar E., Yesilbag Z., Kart-Yasar K. (2020). Severe autoimmune hemolytic anemia in Covid-19 infection. *Mediterranean Journal of Hematology and Infectious Diseases*.

[6] Lazarian G., Quinquenel A., Bellal M. (2020). Autoimmune haemolytic anaemia associated with COVID‐19 infection. *British Journal of Haematology*.

[7] Salle V. (2021). Coronavirus-induced autoimmunity. *Clinical Immunology*.

[8] Chung Y. H., Beiss V., Fiering S. N., Steinmetz N. F. (2020). COVID-19 vaccine frontrunners and their nanotechnology design. *ACS Nano*.

[9] Polack F. P., Thomas S. J., Kitchin N. (2020). Safety and efficacy of the BNT162b2 mRNA covid-19 vaccine. *New England Journal of Medicine*.

[10] Liebman H. A., Weitz I. C. (2017). Autoimmune hemolytic anemia. *Medical Clinics of North America*.

[11] Jäger U., Barcellini W., Broome C. M. (2020). Diagnosis and treatment of autoimmune hemolytic anemia in adults: recommendations from the first international consensus meeting. *Blood Reviews*.

[12] Andrès E., Loukili N. H., Noel E. (2004). Vitamin B12 (cobalamin) deficiency in elderly patients. *Canadian Medical Association Journal*.

[13] Andrès E., Affenberger S., Zimmer J. (2006). Current hematological findings in cobalamin deficiency. A study of 201 consecutive patients with documented cobalamin deficiency. *Clinical and Laboratory Haematology*.

[14] Yeruva S. L., Manchandani R. P., Oneal P. (2016). Pernicious anemia with autoimmune hemolytic anemia: a case report and literature review. *Case Reports in Hematology*.

[15] Sokol R. J., Hewitt S., Stamps B. K., Res C. (1981). Autoimmune haemolysis: an 18-year study of 865 cases referred to a regional transfusion centre. *BMJ*.

[16] Rabinowitz A. P., Sacks Y., Carmel R. (1990). Autoimmune cytopenias in pernicious anemia: a report of four cases and review of the literature. *European Journal of Haematology*.

[17] Olinescu R., Kummerow F. A., Handler B., Fleischer L. (1996). The hemolytic activity of homocysteine is increased by the activated polymorphonuclear leukocytes. *Biochemical and Biophysical Research Communications*.

[18] Ventura P., Panini R., Tremosini S., Salvioli G. (2004). A role for homocysteine increase in haemolysis of megaloblastic anaemias due to vitamin B12 and folate deficiency: results from an in vitro experience. *Biochimica et Biophysica Acta (BBA)-Molecular Basis of Disease*.

[19] Acharya U., Gau J.-T., Horvath W., Ventura P., Hsueh C.-T., Carlsen W. (2008). Hemolysis and hyperhomocysteinemia caused by cobalamin deficiency: three case reports and review of the literature. *Journal of Hematology & Oncology*.

[20] Carli G., Nichele I., Ruggeri M., Barra S., Tosetto A. (2021). Deep vein thrombosis (DVT) occurring shortly after the second dose of mRNA SARS-CoV-2 vaccine. *Internal and Emergency Medicine*.

[21] Angileri F., Légaré S. ., Gammazza A. M., de Macario E. C., Macario A. J. L, Cappello F. (2020). Is molecular mimicry the culprit in the autoimmune haemolytic anaemia affecting patients with COVID-19?. *British Journal of Haematology*.

[22] Gallagher P. G., Tse W. T., Scarpa A. L., Lux S. E., Forget B. G. (1997). Structure and organization of the human ankyrin-1 gene. *Journal of Biological Chemistry*.

[23] Brito S., Ferreira N., Mateus S. (2021). A case of autoimmune hemolytic anemia following COVID-19 messenger ribonucleic acid vaccination. *Cureus*.

[24] Pérez‐Lamas L., Moreno‐Jiménez G., Tenorio‐Núñez M. C. (2021). Hemolytic crisis due to Covid‐19 vaccination in a woman with cold agglutinin disease. *American Journal of Hematology*.

